# Genetic Risk Factors in Early-Onset Nonalcoholic Chronic Pancreatitis: An Update

**DOI:** 10.3390/genes12050785

**Published:** 2021-05-20

**Authors:** Katarzyna Wertheim-Tysarowska, Grzegorz Oracz, Agnieszka Magdalena Rygiel

**Affiliations:** 1 Department of Medical Genetics, Institute of Mother and Child, Kasprzaka 17a, 01-211 Warsaw, Poland; katarzyna.wertheim@imid.med.pl; 2 Department of Gastroenterology, Hepatology, Feeding Disorders and Pediatrics, The Children’s Memorial Health Institute, Aleja Dzieci Polskich 20, 04-730 Warsaw, Poland; grzegorz@oracz.pl

**Keywords:** early-onset nonalcoholic chronic pancreatitis, hereditary pancreatitis, CP, HP, oligogenic disease

## Abstract

Chronic pancreatitis (CP) is a progressive, irreversible inflammatory disorder of the pancreas, which results from interrelations between different genetic and environmental factors. Genetic variants are the primary cause of the disease in early-onset nonalcoholic CP patients. Novel CP-associated genes are continuously emerging from genetic studies on CP cohorts, providing important clues for distinct mechanisms involved in CP development. On the basis of functional studies, the genetic alterations have been sub-grouped into CP-driving pathological pathways. This review focuses on the concept of CP as a complex disease driven by multiple genetic factors. We will discuss only well-defined genetic risk factors and distinct functional pathways involved in CP development, especially in the context of the early-onset nonalcoholic CP group. The diagnostic implications of the genetic testing will be addressed as well.

## 1. Introduction

Chronic pancreatitis (CP) is a long-lasting inflammatory disease starting from acute pancreatitis (AP) (sudden onset) through recurrent acute (more than one episode of acute pancreatitis) up to a progressive, irreversible inflammatory disorder. This disease is associated with periods of exacerbation and remission. It leads to irreversible pancreatic gland destruction with fibrosis. Consequently, permanent morphological changes in pancreas develop, followed by exocrine and endocrine malfunctioning [1,2]. Due to pancreas large functional reserves, exocrine and endocrine insufficiency occurs after about 90% of the gland’s parenchyma is destroyed by the inflammation process. Thus, long-term complications are observed after many years of the disease duration and they are mostly diagnosed in adult patients [1,2].

The prevalence of CP in adults is estimated at 27.4 per 100,000 people in Western Europe, whereas the prevalence of pediatric CP is 5.8 per 100,000 [3]. Causes of early-onset CP (below 35 years old) differ significantly from those described in older adults (alcohol abuse and smoking), and the most frequent are gene mutations, anatomical defects of pancreatic duct (*pancreas divisum*, *ansa pancreatica*), biliary tract diseases (choledocholithiasis, PSC), autoimmune pancreatitis, or lipid disorders (familial hypertriglyceridemia, hyperlipidemias, obesity) [1,4,5,6,7].

Although complex interrelations between environmental and genetic factors are surely involved in adult CP development, hereditary pancreatitis (HP) occurs approximately only in 4% of all CP cases in this group. On the contrary, the most significant role in CP development in early-onset pancreatitis, especially in children, is played by gene variants [4,5,6,8,9].

Genetic variants are important risk factors for early CP and can add specificity to the likely etiology, but they are neither necessary nor sufficient to make a diagnosis. Diagnosis of CP is based on studies of pancreas structure and function as a result of multidisciplinary cooperation of geneticist, gastroenterologist, radiologist and pathomorphologist. According to INSPIRE (International Study Group of Pediatric Pancreatitis: In Search for a Cure) group recommendations, the diagnosis of CP requires 1 of 3 criteria: (1) abdominal pain consistent with pancreatic origin, (2) evidence of exocrine pancreatic insufficiency and (3) evidence of endocrine-pancreatic insufficiency, which were associated with abnormalities on imaging studies (e.g., ductal changes (the irregular contour of the main pancreatic duct or its radicles, intra-ducting-filling defects, calculi, stricture or dilatation) and parenchymal tissues (generalized or focal enlargement, irregular contour cavities, calcifications, heterogeneous echotexture [10])). Imaging studies such as ultrasound, computer tomography (CT scans), magnetic resonance cholangiopacreatography (MRCP), endoscopic ultrasound (EUS) and endoscopic retrograde cholangiopancreatography (ERCP) are most reliable in diagnosing CP. MRCP provides the most accurate visualization of the pancreatic ductal system and has been regarded as the criterion standard for diagnosing chronic pancreatitis [2,11]. Exocrine pancreatic insufficiency (EPI) diagnosis can be made on the basis of several function tests, including fecal fat quantification (considered as the gold diagnostic standard for steatorrhea), fecal elastase-1 (FE-1) and ^13^C-mixed triglyceride breath test (^13^C-MTBT) [12]. At present, histopathology is not necessary to diagnose chronic pancreatitis [2,10,11].

HP develops mainly in childhood. According to current recommendations, the hereditary pancreatitis (HP) is recognized when pathogenic variants in *PRSS1* gene (encoding cationic trypsinogen) are present or when acute pancreatitis or CP of unknown etiology occurred in 2 first-degree relatives or in 3 or more relatives of the second degree in two or more generations [5]. However, when the full criteria for HP family history are not met and the disease has been identified in at least one relative, the diagnosis of familial CP (FCP) is established [8].

**Figure 1:**
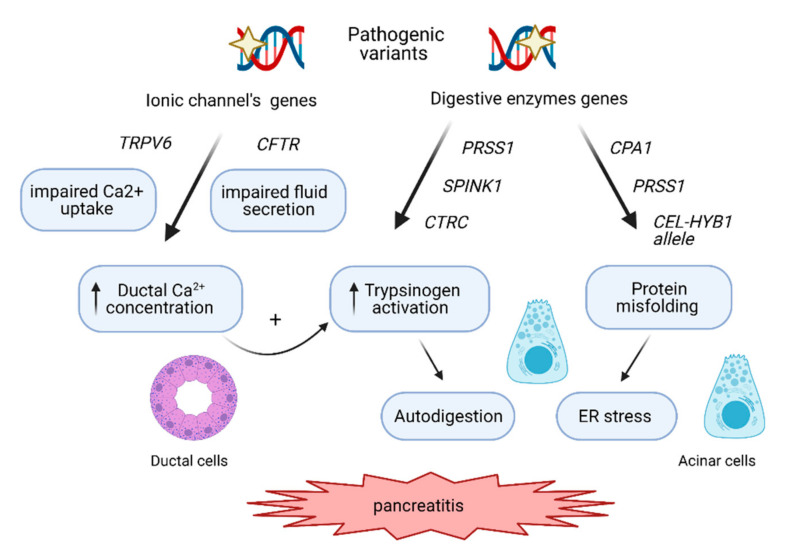
The molecular pathways involved in CP development. The genetic risk in CP is mediated mainly by the trypsin-dependent pathway, where pathogenic variants in *PRSS1* stimulate autoactivation of cationic trypsinogen within the acinar cells, leading to autodigestion of the pancreas. Pathogenic variants in *SPINK1* and *CTRC* disrupt inhibition of cationic trypsinogen activation or its degradation respectively, also contributing to a higher level of trypsinogen activation. The variants in genes such as *CFTR* and *TRPV6* impair ductal secretion and Ca^2+^ uptake, respectively, leading to increased Ca^2+^ concentration in the ductal fluid, which in turn stimulates (auto)activation of trypsinogen. The third mechanisms involve endoplasmic reticulum (ER) stress caused by genetic variants in *CPA*1, *PRSS1* and by *CEL-HYB1* alleles, inducing misfolding of these digestive enzymes and leading to acinar cell damage and inflammatory signaling.

Over the years, besides *PRSS1*, pathogenic variants in *CFTR* (encoding cystic fibrosis transmembrane conductance regulator gene) and *SPINK1* (secretory trypsin inhibitor) genes were linked with increased risk of CP. Nevertheless, up to 30–40% of CP still remains idiopathic [5]. This raised the question of whether other genes are also involved. Finally, recent progress in molecular methodology made it possible to answer this question by identification of the additional genes, which variants confer the CP risk (e.g., *CPA1*, *CTRC*, *TRPV6*, *CEL-HYB1* allele). Hence, currently, the CP is regarded as an oligogenic disease for which development depends on complex integrations between undefined number of genes and different functional pathways (Table 1, Figure 1). Moreover, according to some studies, complex genetic interactions are causally related to even more than 30% of recurrent acute and chronic pancreatitis [13]. 

The aim of this review is to present the current knowledge on the concept of CP as a complex disease caused by multiple genetic factors. We will discuss only well-defined genetic risk factors associated with nonalcoholic CP, with special attention to the early-onset disease group. The diagnostic implications of the genetic testing will be addressed as well. 

## 2. The Molecular Pathways Involved in CP Development 

### 2.1. The Trypsin-Dependent Pathway 

The identification of activating pathogenic variants in cationic trypsinogen gene *PRSS1* (a precursor of major pancreatic digestive enzyme) in hereditary pancreatitis supported the classical theory of pancreatitis as an autodigestive disease [14]. Pathogenic variants in *PRSS1* stimulate autoactivation of cationic trypsinogen within the pancreas, triggering the activation of pancreatic digestive zymogenes cascade, which leads to autodigestion of the pancreas. Therefore, most of heterozygous pathogenic variants of *PRSS1 *are regarded as a cause of hereditary pancreatitis with autosomal dominant inheritance [15]. The genetic studies of different forms of this disease such as familial CP (FCP) and idiopathic CP (ICP) have identified pathogenic variants in other genes, including secretory trypsin inhibitor (*SPINK1*) and chymotrypisinogen C (*CTRC*) [16,17,18]. SPINK1 inhibitor is responsible for protecting the pancreas from premature, pathological trypsin activation, while CTRC is involved in trypsinogen degradation. Thus, pathogenic variants in *SPINK1* and *CTRC* genes disrupt inhibition of cationic trypsinogen activation or its degradation, respectively [19].

### 2.2. The Misfolding-Dependent Pathway 

Another pathway involved in CP genetic risk is endoplasmic reticulum (ER) stress. This mechanism is independent of the trypsin and is caused by misfolding of digestive enzymes due to genetic variants [20], which leads to diminished secretion, intracellular retention and endoplasmic reticulum stress. Consequently, acinar cell damage may occur as well as induction of inflammatory signaling. 

Up to now, the best identified misfolding variants increasing CP risk or causing hereditary pancreatitis have been discovered in *PRSS1* and *CPA1* genes [21,22]. More recently, it was suggested that another genetic alteration: *CEL* and *CELP* hybrid allele (known as *CEL-HYB1*), may be associated with the misfolding-dependent pathway [23].

### 2.3. The Ductal Pathway

The third pathomechanism related to pancreatitis genetic risk is a ductal pathway which is caused by genetic variants in the genes encoding proteins involved in ductal secretion. So far, variants in two genes encoding transmembrane ionic channels: *CFTR* (cystic fibrosis transmembrane conductance regulator) and the *TRPV6* (the transient receptor potential cation channel, subfamily V, member 6), seem to be involved. It is speculated that the variants of these genes cause the alteration of the ductal fluid Ca^2+^ concentration and, hence, increase ductal Ca^2+^ levels that eventually would stimulate autoactivation of trypsinogen [24,25]. 

**Table 1:** Pathways associated with genetic variants involved in chronic pancreatitis development.

Gene (#OMIM)	Protein	CP Etiology	Genetic Variants #	Mechanisms	Pathway
*PRSS1* (276000)	Cationic trypsinogen	HP, rarely sporadic CP	p.Asn29Ile, p.Ala16Val	• increasing CTRC-dependent stimulation of autoactivation	Trypsin-dependent
			p.Arg122His, p.Arg122Cys, p.Val39Ala	• increase trypsinogen autoactivation by inhibition of CTRC-dependent trypsinogen degradation	
			Rare variants (e.g., Asp19Ala, p.Asp21Ala)	• directly stimulate autoactivation of trypsinogen independently of *CTRC* function	
			Rare variants (e.g., p.Leu104Pro, p.Arg116Cys)	• reduced trypsinogen secretion intracellular retention and elevated ER stress markers	Misfolding-dependent
*SPINK1* (167790)	pancreatic secretory trypsin inhibitor	ICP,	p.Asn34Ser	• unknown	Trypsin-dependent
			c.194+2T>C, and rare missense or nonsense variants	• diminished production or secretion of protective trypsin inhibitor	
*CTRC* (601405)	chymotrypisinogen C	ICP	p.Lys247_Arg254del, p.Arg254Trp, p.Val235Ile	• loss of protein function and reduced expression, leading to inefficient trypsinogen degradation	Trypsin-dependent
*CPA1* (114850)	carboxypeptidase A1	HP and ICP	p.Asn256Lys, p.Ser282Pro, p.Arg382Trp	• misfolding affecting secretion and intracellular retention, leading to elevated ER stress	Misfolding-dependent
*CEL*-*HYB1* (*CEL*: 114840)	Recombinant of carboxyl ester lipase and carboxyl ester lipase pseudogene	ICP	CEL-HYB1 allele (CEL and CELP recombinant) ((p.Thr488-Ile548 haplotype)	• proteotoxic protein-misfolding	Misfolding-dependent
*CFTR* (602421)	cystic fibrosis transmembrane conductance regulator	ICP	p.Phe508del and other variants severely affecting CFTR expression and activity, p.Arg117His	• abnormal ductal fluid levels, consistency and pH due to disrupted ion transportation, probably leading to increased Ca^2+^ concentration	Ductal
*TRPV6* 606680	transient receptor potential cation channel, subfamily V, member 6	ICP and familial CP	p.Glu575Lys, p.Arg345His, p.Arg483Trp	• defect in Ca^2+^ transport; the dysfunctional variants may cause elevated ductal Ca^2+^ concentration	Ductal

CP—chronic pancreatitis, HP—Hereditary pancreatitis, ICP—idiopathic chronic pancreatitis, familial CP, # examples of most frequent variants; full list of variants for *PRSS1*, *SPINK1*, *CTRC,* and *CPA1* may be found in http://pancreasgenetics.org/ (accessed on 14 May 2020); *CFTR* and *CEL* variants—see references cited in the main text, *TRPV6*, according to Massamune et al. and Zou et al. [26,27].

## 3. The Genetic Variants Associated with CP

### 3.1. PRSS1

The most frequently found *PRSS1* pathogenic variants belong to the trypsin-dependent pathway and cause hereditary pancreatitis with incomplete penetrance. The presence of these variants encourages cationic trypsinogen activity by different mechanisms, i.e., direct autoactivation stimulation, increase of CTRC-mediated processing of the activation peptide or by reduction of CTRC-dependent degradation [25]. The most common *PRSS1* variants worldwide are p.Arg122His and p.Asn29Ile, which are present in 60–80% of HP cases [6,28,29]. The *PRSS1* pathogenic variants such as p.Arg112Cys, p.Ala16Val and p.Glu79Lys are less frequent and p.Asp19Ala, p.Asp21Ala, p.Asp22Gly and p.Lys23Arg are rare variants [6,30]. The distributions of these *PRSS1* variants may differ between different countries. For example, in Polish children with HP (age at onset around 8.5 years), the most common pathogenic variant is p.Arg122His (34%), which is consistent with the other data [28,31,32]. However, the second most prevalent pathogenic variant is p.Arg122Cys (27%), for which frequency is much lower in other cohorts and ranges from 0% to 1.5% [28,31,32,33]. The p.Asn29Ile pathogenic variant, the second most frequent in the world, in the Polish patients was observed in only 12% of the cases, and the very rare p.Glu79Lys pathogenic variant worldwide was found in as many as 7% of the cases. 

Functionally, the p.Arg122Cys and p.Arg122His variants prevent CTRC-dependent trypsinogen degradation, whereas the p.Asn29Ile affects trypsinogen biochemistry, strengthening the process of its autoactivation [34]. Similarly, in case of variant p.Ala16Val, autoactivation is enhanced. In this case, the activation peptide of trypsinogen to CTRC-mediated processing is more sensitive [34,35]. However, the p.Ala16Val variant shows reduced disease penetrance compared to p.Arg122His, which may be explained by lower trypsin levels generated by the p.Ala16Val variant. 

Rare *PRSS1* variants (p.Asp19Ala, p.Asp21Ala, p.Asp22Gly, p.Lys23Arg and p.Lys23_Ile24insIleAspLys) have an impact on the cationic trypsinogen activation peptide and hence stimulate autoactivation in a CTRC-independent way [36,37].

The distinctive mechanism based on both increased trypsinogen activation and secretion was observed in case of the *PRSS1-PRSS3P2* conversion allele found in two unrelated patients in Poland and Germany [38]. The converted region between exon 3 of the *PRSS1* and *PRSS3P2* contained three non-synonymous amino acid changes, p.Ser115Thr, p.Arg116Pro and p.Arg122His. The pathogenic effect of the *PRSS1-PRSS3P2* conversion allele is due to increased trypsinogen activation and secretion, attributed to p.Arg122His and p.Arg116Pro variants, respectively. 

The *PRSS1* variants associated with the misfolding-dependent pathway are rare. They cause reduced secretion, intracellular retention and elevated ER stress markers. The p.Leu104Pro and p.Arg116Cys were detected in HP with incomplete penetrance, whereas p.Cys139Phe, p.Cys139Ser and p.Gly208Ala are mostly associated with sporadic CP [20,22]. 

The *PRSS1* variants presumed to cause CP, such as p.Asp100His, p.Cys139Phe, and p.Lys92Asn, p.Ser124Phe and p.Gly208Ala, lead to diminished trypsinogen secretion in in vitro studies, indicating the misfolding-dependent pathway as a probable pathomechanism in this case [39].

### 3.2. SPINK1 

There are two major molecular mechanisms conferring the *SPINK1*-related elevated risk of chronic pancreatitis. Both of them, reduced expression of mRNA and protein, cause diminished production of protective trypsin inhibitor [16].

The most frequent variant found in *SPINK1* is p.Asn34Ser, which according to meta-analysis studies was detected in 9.7% of CP alleles (469/4842) compared to 1% of control alleles (96/9714) [40]. In Polish early-onset nonalcoholic CP patients, variants in *SPINK1* have a frequency of 8–28% [6,8,41]. In European populations, the p.Asn34Ser increases CP risk about 10–15-fold [42]. The exact mechanism of p.Asn34Ser molecular pathophysiology remains unclear. According to recent studies by Szabó et al., p.Asn34Ser variant does not impact trypsin inhibition or enhanced SPINK1 degradation [43]. Moreover, only 1% of the p.Asn34Ser carriers develop symptoms of acute or chronic pancreatitis, indicating that other risk factors are involved [44,45]. The haplotype context of p.Asn34Ser was hypothesized to be one of them [43]. 

Moreover, it has not been established yet whether heterozygous p.Asn34Ser, when present together with additional genetic or environmental risk factors, should be treated as a disease-modifying or causative factor [43]. It should be noted, however, that no p.Asn34Ser homozygote has ever been found in a healthy population and patients with homozygous p.Asn34Ser develop severe symptoms of CP [18,46]. Therefore, it is assumed that *SPINK1* p.Asn34Ser heterozygous variants contribute to CP, whereas p.Asn34Ser homozygous are causative of CP [47,48].

Unlike in the case of p.Asn34Ser, the pathomechanism of another frequent *SPINK1* variant—the intronic c.194+2T>C—is rather clear as it leads to skipping of exon 3 [16,49,50]. Besides, around 60, mainly rare or private, variants in *SPINK1* have been reported, according to the HGMD database. Although in case of certain variants miscellaneous mechanisms were postulated to affect SPINK1 activity, the reduced expression and/or secretion seem to be mostly involved [16]. 

### 3.3. CTRC 

The pathogenic variants of *CTRC* generally cause loss of protein function [51,52]. For example, the p.Lys247_Arg254del variant makes the protein inactive and easily degraded, and the p.Arg254Trp leads to degradation by trypsin [51]. In adult CP patients, the p.Lys247_Arg254del and the p.Arg254Trp are rare, found with frequency of 0.1% to 1.5% and 1.2% to 2%, respectively (and with 5-fold increased CP risk on average) [17,18,53]. In early-onset (pediatric) CP patients, however, the p.Lys247_Arg254del (5.3%) and p.Arg254Trp (4.6%) variants are relatively frequent (combined frequency of 9.6%), as demonstrated in Polish CP children [54]. In this cohort, p.Arg254Trp and p.Lys247_Arg254del are associated with 19-fold and 5-fold increased CP risk compared to controls, respectively. 

There is a frequent p.Gly60Gly *CTRC* heterozygous variant found in 30–40% of CP patients, including early-onset CP patients [17,54,55]. In the early-onset patients, CP risk is increased by 2.5-fold in case of p.Gly60Gly heterozygosity and by 24-fold in p.Gly60Gly homozygosity [54]. The mechanism associated with p.Gly60Gly is unclear, however, some data may suggest reduced *CTRC* mRNA expression, possibly caused by altered pre-mRNA splicing [30].

### 3.4. CPA1 

Digestive carboxypeptidases are pancreatic metalloproteases, which hydrolyze C-terminal peptide bonds in dietary polypeptide chains. In 2013, Witt et al. reported that variants in the *CPA1* (carboxypeptidase A1) gene are strongly associated with CP (OR ~ 25), and, in particular, with sporadic cases of CP with early onset (OR ~ 80) [21]. It was shown that *CPA1 *variants confer increased CP risk by misfolding affecting secretion, causing intracellular retention and inducing ER stress, rather than affecting trypsin activity. Nevertheless, it has not been analyzed if *CPA1* variants are causative risk factors for the familial or hereditary disease. However, a study by Kujko et al. shed light on this issue, showing that the novel *CPA1* variant p.Ser282Pro co-segregated with pancreatitis in two Polish families with autosomal dominant hereditary pancreatitis [56]. The age of onset for index patients in these two families was 17 and 12 years. The p.Ser282Pro *CPA1* variant causes protein retention and degradation (indicative for misfolding), leading to ER stress. Interestingly, the same mechanism has also previously been described for the p.Leu104Pro *PRSS1* variant [57]. 

Importantly, recently, a study on a mouse model for the misfolding-dependent pathway was published. The authors constructed the knock-in mice harboring the most frequent *CPA1* human variant: p.Asn256Lys, and demonstrated development of spontaneous and progressive CP together with ER stress symptoms in their pancreas [58].

### 3.5. CEL-HYB1 Allele 

The *CEL* gene encodes a carboxyl ester lipase (previously known as bile salt-stimulated lipase), which is a digestive enzyme produced in pancreas acinar cells and secreted into the small intestine. The CEL protein is activated by bile salts and is involved in hydrolysis and absorption of cholesterol and lipid-soluble vitamin esters [59]. 

In 2015, Fjeld et al. characterized a hybrid allele *CEL-HYB* (currently referred to as *CEL-HYB1*), which originates from a non-allelic homologous recombination between CEL and its pseudogene CELP. The average population frequency of *CEL-HYB1* recombinant was 0.5–1%, while in a group of German and French idiopathic CP, it was 5-fold overrepresented [23]. The cell culture experiments revealed intracellular retention and poor secretion of the hybrid protein. It was proposed that the *CEL-HYB1* allele may increase CP risk through the misfolding-dependent pathway [23]. However, the study of Zou et al. showed that the *CEL-HYB1* allele is not present in three independent CP Asian cohorts—from China, Japan and India. Hence, the authors came up with an idea that the *CEL-HYB1* allele could be a European ethnicities-specific CP risk factor [60]. Unexpectedly, another European replication study on Polish CP pediatric cohorts failed to find any significant difference in the *CEL-HYB1* allele frequency between CP patients and controls (4.8% vs. 2.4%, *p* = 0.16). Moreover, the distribution of alleles in the Polish control group was higher than in German, French and Norwegian controls: 2.4% (12/500) vs. 0.7–1%, 0.7% and 0.3%, respectively [22,61,62].

In 2020, further studies with German and French patients revealed the presence of two amino acid substitutions in *CEL-HYB*, p.Thr488-Ile548, in CP cases but not in controls. On the contrary, the frequency of another, similar variant, thep.Thr488-Thr548, was not significantly different between patients and control groups. It was suggested that the *CEL-HYB1* allele harboring the p.Thr488-Ile548 haplotype may be responsible for increased risk of CP. Surprisingly, the functional assays on p.Thr488-Ile548 and p.Thr488-Thr548 variants revealed that both variants show similar pathogenicity and cause proteotoxic protein-misfolding [63]. The discordance between genetic and functional data in case of the p.Thr488-Thr548 haplotype indicates that the role of *CEL-HYB1* allele variants in CP development remains unclear and requires further investigation [63].

### 3.6. CFTR 

The *CFTR* gene encodes the cystic fibrosis transmembrane conductance regulator, the ABC family membrane transport protein channel, localizing in the apical plasma membrane of epithelial cells [64]. 

Pathogenic variants in the *CFTR* gene are responsible for cystic fibrosis but are also risk factors of *CFTR*-related disorders. CF patients may clinically present with exocrine pancreatic insufficiency and, rarely, pancreatitis. Studies on cystic fibrosis indicate, however, that not all *CFTR* variants are correlated with pancreas insufficiency [65,66,67].

CFTR is crucial for the maintenance of proper ductal fluid levels and consistency and plays a role in regulating bicarbonate (HCO_3_^−^) secretion [68]. Thus, loss of CFTR function may cause decreased bicarbonate secretion and impaired ductal flushing [24,69]. It has been suggested that the decrease in bicarbonate secretion may prolong the time of zymogens transport to the duct and decrease pH, and thereby promote the autoactivation of trypsinogen. Activated trypsin would further inhibit CFTR channel activity and bicarbonate secretion, leading to intraductal acidosis. Acidosis would further enhance trypsinogen autoactivation, which could induce development of the pancreatitis [69]. This mechanism, however, may be particularly important in HP, where the mutated trypsinogens (i.e., with the p.Arg122His in PRSS1) are easily autoactivated.

Mutations in the *CFTR* gene are divided into six classes, depending on the severity of their impact on the protein synthesis and activity [70]. In general, compound heterozygous state for two severe mutations leads to cystic fibrosis, while when at least one allele harbors the mild *CFTR* allele, the *CFTR*-related disorder occurs. Hence, being a compound heterozygote for one severe and one mild *CFTR* mutation is considered as a strong risk or even causative factor for chronic pancreatitis [71]. Moreover, it has also been proven that p.Phe508del heterozygosity confers a 2.5 times increased risk for CP, while the mild p.Arg117His variant increases risk by about 4-fold [47]. There are over 2000 variants in the *CFTR* gene and involvement in pancreatitis or other CFTR-related disorders of several of them remains controversial [71]. For example, it has been postulated that certain non-CF-causing CFTR variants (e.g., p.Arg75Gln, p.Leu997Phe) leading to a selective, bicarbonate transportation defect in CFTR channel function may be risk factors for chronic pancreatitis [72]. While functional measurements seem to support this finding, a genetic association between these variants and chronic pancreatitis cannot be confirmed [47,53].

### 3.7. TRPV6

TRPV6 is a tetrameric, calcium-selective channel participating in epithelial Ca^2+^ (re)absorption [73]. The TRPV6 is predominantly expressed in ductal cells, which suggests a role in controlling calcium concentration of pancreatic juice, however its precise function is not yet fully discovered [74]. 

In 2020, two research groups showed that loss of function variants of TRPV6 are associated with risk of chronic pancreatitis in patients from Japan, Germany, France and China [26,27]. During our research towards identification of novel CP susceptibility genes using a whole exome sequencing approach, we also found overrepresentation of the novel defective *TRPV6* variants in Polish CP pediatric patients (data unpublished, [75]). These findings taken together indicate that *TRPV6* is a novel CP susceptibility gene.

In nonalcoholic early-onset CP, *TRPV6* variants contribute to disease pathogenesis in 1.5–4% of the patients [26,27]. Since it is speculated that TRPV6 plays a role in Ca^2+^ removal from the ducts, the loss-of-function variants could cause elevated ductal Ca^2+^ concentration, which would stimulate autoactivation of trypsinogen and increased trypsin activity. However, the exact mechanism of how *TRPV6* variants alter pancreatic Ca^2+^ balance requires further investigation. Probably, the cumulative genetic handicap is necessary to develop CP, which is supported by the fact that *TRPV6* variants often coexist with pathogenic variants in other CP susceptibility genes such as *SPINK1* and *CFTR*, according to Masamune et al. [26].

## 4. Complex Genetic Interactions

It is known that CP may develop due to simultaneous presence of variants in different recessive genes [76]. The paradigm of gene–gene interactions appears to be a co-existence of a variant increasing the recurrent trypsin activation risk (e.g., in *PRSS1*, *CFTR* genes), together with a variant protecting the pancreas from active trypsin (e.g., *SPINK1*, *CTRC* genes) or chronic inflammation. Such interactions are well-documented in patients who carry both *SPINK1* and *CFTR* variations. Pancreatitis risk increases approximately 60-fold by having the heterozygote genotype in *SPINK1* and *CFTR* (one variant in one allele of *SPINK1* and one variant in one allele of *CFTR*), whereas the CP risk increases up to 900-fold by having a *CFTR* compound heterozygote genotype (two heterogeneous variants) and the *SPINK1* p.Asn34Ser variant in one allele [67,77]. Coinheritance of the *SPINK1* p.Asn34Ser in one allele and at least one abnormal allele in the *CFTR* accounts for 1.5–7.7% of the cases, depending on the population analyzed. *Trans*-heterozygotes of *SPINK1* p.Asn34Ser and *CTRC* (defect in one allele of *SPINK1* and one allele of *CTRC*) were detected in 1% of German CP patients and in 2.3% of young French ICP patients [17,18]. 

While the *CFTR/SPINK1 trans*-heterozygosity has a synergistic effect [72], the *SPINK*1/*CTRC trans*-heterozygosity is rather connected with an additive or a multiplicative impact, as those genes are involved in the same pathway [78]. Similarly, *trans-*heterozygosity of *TRPV6* and *CFTR* variants may have an additive effect (two genes belong to the ductal pathway), whereas in case of *TRPV6*/*SPINK1, trans*-heterozygosity may show a synergistic effect [24]. 

## 5. Diagnostic Implications

According to the European guidelines, all patients with a family history or early-onset disease should be offered genetic testing for associated genes [2]. Since alcohol abuse is the predominant cause of the disease in up to 70% of adult cases, genetic screening for every CP patient is not recommended. In pediatric cases, the genetic screening should be offered for the patients with a second episode of idiopathic AP or first episode of idiopathic AP, and a family history of AP or CP. In these patients, the analysis of the presence of the pathogenic variants in *PRSS1*, *CPA1*, *SPINK1*, *CTRC* and *CFTR* genes as well as testing for the CEL gene pathogenic hybrid allele is recommended [11]. Hereditary pancreatitis associated with mutations in *PRSS1*, especially p.Arg122His, could considerably increase the risk of pancreatic adenocarcinoma (87 times more likely at age 55) [79]. Since more than 95% of pancreatic cancers are diagnosed at a later stage, the 5-year survival rate remains only 6% [80]. Therefore, in Poland, the USA and many other countries, pancreatic cancer screening (neoplasm markers, EUS and/or ultrasound/yearly) is recommended for all CP patients ≥ 35 years old who carry pathogenic variants in *PRSS1* [81,82]. 

In recent years, the knowledge about molecular aspects of chronic pancreatitis has markedly increased, and therefore, the current guidelines need further updates. In this respect, the genetic screening of the *TRPV6* gene should be included for early-onset CP patients. Besides, in the light of the novel data, screening towards functionally defective *CEL-HYB1* allele variants seems to be more appropriate than investigating the presence of the allele itself. Moreover, there are still several questions unanswered with the respect to the multifactorial etiology of idiopathic CP. Here, both molecular diagnostic and genetic counseling are becoming more important and require further consideration.

In case of probands’ relatives, molecular testing is a basis for establishing a carrier status, which enables not only CP risk assessment, but also implementation of preventive actions or, in milder cases, even early diagnosis [83,84]. While in autosomal dominant HP due to *PRSS1* or *CPA1* pathogenic variants the case is rather clear, it becomes much more complicated in idiopathic CP when other genes are involved. The combinations of genetic factors may be epistatic, while others are additive, therefore the genetic risk assessment should be handled on a case-by-case basis. Unfortunately, no systematic approach is available to predict the effects of most of these complex genotypes [13]. Since it is widely accepted that the effect of the variant in one CP-connected gene is affected by the presence of a variant in another gene, the genetic risk assessment becomes challenging. Since no systematic approach has been elaborated, in case of the complex genotypes, individual assessment for each person is needed [13]. 

In 2013, Masson et al. proposed a clinically useful classification of variants and genotypes found in CP patients, which were divided into three categories: causative, contributory or neutral. The factors considered in this assessment include: allelic frequency in healthy and CP groups, functional defects, the relative importance of their associated genes in the pathogenesis of chronic pancreatitis and intergenic interactions, when applicable [47].

More recently, another group proposed a scoring system, which enables the classification of variants into four categories: (1) established risk alleles, (2) likely risk alleles, (3) uncertain risk alleles and (4) benign variants. This categorization of variants is based on the results of in vitro experiments, functional and epidemiological data and in silico predictions. Importantly, the authors elaborated a very clear scoring system, which enables rather unequivocal classification. This scoring system was used to evaluate the status of all variants known at the time in *SPINK1* (*n* = 56) and *CTRC* (*n* = 87). In the case of *SPINK1,* 25/56 variants were scored as established risk alleles, 8/56 as likely risk alleles, 17/56 as uncertain and 6/56 as benign. In the case of *CTRC*, the quantity of individual groups was: 24/87, 4/87, 22/87 and 37/87, respectively [84]. 

These results, otherwise very important and useful in clinical settings, also illustrate the complexity of interpretational issues in CP genetics, which still involves not only establishing of individual variant status, but also assessment of intergenic relations. 

## 6. Conclusions

Nonalcoholic chronic pancreatitis is a complex disease which may be caused by multiple genetic factors. Genetic testing for CP patients with unknown etiology is recommended by analyzing major CP-associated genes such as *PRSS1*, *CPA1*, *CTRC*, *CFTR, SPINK1* and *TRPV6*, especially in early-onset cases and after the other major causes of the disease have been excluded. The diagnostic utility of the *CEL-HYB1* allele and its variants requires further research. 

Genetic counseling both before and after molecular testing is strongly recommended in all patients. Importantly, CP patients having genetic variants should be carefully followed by the clinicians since they may have a high risk of developing further medical complications such as pancreatic exocrine insufficiency, diabetes mellitus and pancreatic cancer [85]. 

The progress and availability of large-scale molecular methods enable unprecedented insight into hereditary pancreatitis genetics. Hence, proof that variants in other genes contribute to early-onset nonalcoholic chronic pancreatitis will surely emerge. Clearly, the genetic guidelines for clinical use are needed to help to stratify the risk of CP associated with different genetic factors.
